# Possible relation between psychosis and the unconscious: a review of “The Unconscious,” by Freud

**DOI:** 10.3389/fpsyg.2015.01001

**Published:** 2015-07-15

**Authors:** Jacqueline de Oliveira Moreira, Carlos R. Drawin

**Affiliations:** ^1^Extended General Practice in Health, Department of Psychology, Pontifical Catholic University of Minas GeraisBelo Horizonte, MG, Brazil; ^2^Philosophy, Federal University of Minas Gerais and Jesuit School of Philosophy and TheologyBelo Horizonte, MG, Brazil

**Keywords:** psicose, unconscious, Freud, Freudian Theory, metapsychology

## Abstract

This review intends to present some elements of the Freudian thinking on psychosis, focusing on the relations between psychosis and the unconscious. The unconscious phenomena which episodically cross the neurotic individual are massively and continuously shown on psychosis. The psychotic individual appears to be constantly invaded by the *other*, like a strange person, which bursts inside of him/her and presents itself as a threat to the process of construction of this person’s identity. But what is the relation between the unconscious and psychosis in the Freudian text? It could be hypothesized that the psychotic individual may be invaded by a pulsating unconscious which demands a symbolic mediation. This reveals the importance of associating verbal construction to medication in cases of psychosis.

It is known that Freud’s professional background was in Neurology, under the orientation of the intensely anatomic 19th century German medicine (although Freud was Austrian, his most influential teachers were representative of German neurophysiologic tradition and supported the scientific presuppositions of the Berlin Physicalist Society). Therefore, Freud’s medical practice did not take place in psychiatric institutions and his oeuvre is not extensively dedicated to the issue of psychosis. Nevertheless, it is undeniable that Freud’s efforts to theorize psychic pathologies offer relevant contribution for a dynamic comprehension of psychosis, which had been systematically investigated by Emil Kraepelin. One should not fail to mention the fact that Freud made an appropriation of the clinical material collected by Bleuler and Jung in order to corroborate the hypothesis which suggests that, in cases of schizophrenia, words may be treated as things (D*inge*), approximating schizophrenic speech and neologisms to the “organ speech,” which is a characteristic of hypochondria.

Freud’s interest in psychosis can be noticed since the very beginning of his theoretical work, as, motivated by his investigations regarding neurosis’ etiology, he compared characteristics of neurosis to classic psychotic pictures, such as melancholy (Freud and Draft, 1895a) and paranoia (Freud and Draft, 1895b). This comparative and etiological investigation method appeared also in seminal articles such as “The Neuro-psychosis of Defense” (Freud, 1894) and“Further Remarks on the Neuro-psychosis of Defense” (Freud, 1896). At that time, Freud intensely dedicated himself to unveil the psychic mechanisms of phobias, obsessions, and, more specially, of hysteria. The studies conducted during this period led to two major theoretical formulations: “The Interpretation of Dreams” (Freud, 1900) and “Three Essays on the Theory of Sexuality” (Freud, 1905). Nonetheless, clinical phenomena were obscure, and matted, demanding, thus, well-thought-out theoretical criteria for grounding psychopathological classifications. It is importante to mention that the matter of transference is also taken into consideration in the lacanian thought. In *On a Question Preliminary to any Possible Treatmentof Psychosis* (Lacan, 1957–1958), Lacan argues that there is a possible treatment of psychosis. Further discussions on this, however, would extrapolate the proposal of this article, which is to review a Freudian text.

Firstly (Freud, 1985–1900), Freud accepts the distinction between neurosis and psychosis elaborated by German psychiatry, but his focus was directed toward the distinction between “current neuroses” (originally translated to English as “actual” neuroses), originated from sexual somatic dysfunctions, and “psychoneuroses” originated from psychic conflicts. It is important to mention, here, Freud’s classic reading of an autobiographic report: the Schreber case (Freud, 1911b), which described a case of paranoia. Later, in a moment in which Freud faced difficulties to obtain a wider theoretical synthesis (1914–1920), he deepened the distinction between neurosis and psychosis through the discovery of the narcissistic constitution of psyche. This was the point in which he elaborated core texts, such as “On Narcissism: An Introduction” (Freud, 1914) and the “Papers on Metapsychology” (Freud, 1915–1917), that showed a deep connection between theory, clinical work and psychopathology. Thus, the 1915 classification maintains the distinction between “current (actual) neuroses” and “psychoneuroses,” and also observes the differences between two types of psychoneurosis: “transference neuroses” (obsessional neurosis, hysteria, and phobia) and the “narcissistic neurosis.” The firsts preserve the libidinal connection with objects, allowing the establishment of transference in the analytical process. In the case of the latter, libidinal connection is compromised, and the libido originated from objects returns to the Ego and is crystallized in a narcissistic position, limiting the individual’s access to external reality.

In his final psychopathological classification (Freud, 1924a,b), Freud retakes the consecrated designation of “psychosis,” which now included pictures of schizophrenia and paranoia, leaving the term “narcissistic neurosis” to refer only to pictures of melancholia. In the article “Neurosis and psychosis” (Freud, 1924a) and “The Loss of Reality in Neurosis and Psychosis” (Freud, 1924b), the thinker distinguishes these two concepts using the hypotheses proposed 1 year earlier, in “The Ego and the Id” (Freud, 1923): “Neurosis and Psychosis,” for instance, proposes a topic differentiation based on “The Ego and the Id” and, thus, proposes a third picture, namely the “narcissistic neuroses.” While transference neurosis presents a conflict between ego and id and the psychoses present a conflict between the ego and the external world, the narcissistic neuroses consist on a conflict between ego and superego.

This brief introduction to the long trajectory of Freudian investigation does not make justice to its subtleties and theoretical oscillations, but may be useful for the focalization of a moment of crucial transformation in his comprehension of psychosis. This moment is represented mainly by two exceptionally fecund metapsychological texts (written almost simultaneously, but published within a 2 years interval): “The Unconscious” (Freud, 1915a) and “Mourning and Melancholia” (Freud, 1917).

It is known that, during the First Topography (Freud, 1900–1920), Freud manifested great interest in the “Psychoneuroses of Defense.” However, after acknowledging the existence of narcissism, he started using the term “Narcissistic Neurosis” to define the clinical experience of psychosis, based on metapsychology, and psychopathology. The concept of transference represents, in this perspective, the defining criteria for the two types of psychoneurosis, namely, transference neurosis and narcissistic neurosis. In the narcissistic neuroses with reflux of libido, there is a barrier for an effective process of transference, that is, the transference bond with the analyst does not occur, and the libido gets repressed. Nonetheless, this review article focuses, mainly, in presenting the Freudian thoughts on psychosis and its articulation with the concept of unconscious and the issue of the other, explaining, thus, the focus on “The Unconscious” (Freud, 1915a).

The phenomena of the unconscious episodically cross the neurotic individual and announce, through various symptoms, dreams, and faulty acts, the discontinuity in the egoic functioning. In the psychotic individual’s case, these phenomena are even more common, appearing continuously and massively in their lives. These individuals appear to be constantly invaded by the *other* which bursts inside of him/her and often presents itself as a threat to the process of construction of this person’s identity. In the aforementioned 1915 article, Freud develops an association between the schizophrenic’s conscious speech and the oneiric processes through which this individual goes. He concluded that the psychic processes of psychosis are subject to the primary process, to a free flow of energy which announces a regression to the hallucinatory satisfaction of desire (*Wunsch*): in the case of schizophrenia, the individual regresses to auto-erotism; and, in the case of paranoia, he/she regresses to primary narcissism.

This fixation with the primary process, which is ruled by the principle of pleasure, produces a schism with reality that compromises a wide range of gains originated by the principle of reality. Skills such as attention, judgment, and rational thinking are lost when there is a schism with reality in the psychotic mind. According to Freud, this is a difference between neurotic and psychotic individuals: while the first do not repudiate reality (they only ignore it), the latter not only repudiate it, but try to replace it (Freud, 1924b).

In this perspective, the author notices that “all observers have been struck by the fact that in schizophrenia a great deal is expressed as being conscious which in the transference neuroses can only be shown to be present in the *Ucs*. [Unconscious] by psycho-analysis^1^” (Freud, 1915b). This statement led to the proposition of the hypothesis that, in narcissistic psychoneuroses (psychoses), one can witness the “unconscious open to the sky,” since words explicitly and directly reveal unconscious content. However, Freud also shows that the character of strangeness of schizophrenia results from the predominance of word-representation (*Wortvorstellung*) over thing-representation (*Sachvorstellung*). The second proposition seems to contradict the hypothesis which simply defines psychosis and unconscious. Word-representation, articulated with thing-representation, belongs to the preconscious and is what makes communication with the outside world possible. In the unconscious, on the other hand, this articulation between word-representation and thing-representation does not exist: only thing-representation is observed. Therefore, it can be **hypothesized** that, if in psychosis there is a domain of word-representation over thing-representation, and the first is absent in the unconscious, it is not correct to affirm that psychosis is a direct expression of the unconscious, or the unconscious “open to the sky.”

Nonetheless, a doubt soon came to Freud’s mind: how can we affirm the presence of the unconscious in psychosis if there has been a rejection of castration (*Verwerfung*), and repression has not taken place? On the one hand, the unconscious system is ruled by the primary process. Since the psychotic individual’s psychic apparatus is also ruled by the primary process, with a domain of the principle of pleasure over the principle of reality, it can be **hypothesized** that there is a predominance of the unconscious system in cases of psychosis. On the other hand, the theory of psychic apparatus, developed in Freud’s First Topography, had established a structural relation between the unconscious and repression. Is it possible to conclude, thus, that this affirmation of the First Topography is incorrect and the immediate association between unconscious and repression should be abandoned? It can be noticed that, by facing the issue of psychosis, Freud was obligated to review and question metapsychology. He, then, introduced the concept of Id as a possible response for such crucial matters.

It appears, hence, that in psychosis repression keeps on operating as a primal repression (*Urverdrängung*), but a later incidence or proper repression and its effect of psychic splitting would also be seriously affected. In this perspective, the unconscious in psychosis can be understood in a dynamic and economic manner, but not in a topic way. There is no structural splitting in the two great systems, which means that the preconscious does not censor the unconscious contents and the borders are rather fluid. Nevertheless, a clear manifestation of this structural absence of barriers, i.e., limits that circumscribe different systems, only occurs after a first episode in which the individual is taken over by the *other*, by a stranger who inhabits his/her body. Therefore, it is very common for the psychotics not to recognize themselves and speak of themselves in the third person.

But how would this refusal of reality occur in the perspective of the First Topography? It is possible to suggest that the perception of external objects results from a combination of word-representation and thing-representation, an articulation which takes place in the preconscious system. In psychosis, the perception and interpretation of reality fail, since, instead of being libidinously invested in the preconscious system, the thing-representation occurs in the unconscious. This means that the preconscious system is invaded by the unconscious in a search for primitive forms of libidinous satisfaction, such as hallucination. It is more of an invasion performed *by* the unconscious than a topic regression *to* the unconscious, although it can be argued that there is a temporal regression to archaic forms of egoic organization, such as auto-erotism, and primary narcissism. Temporal regression and primitive forms of satisfaction help the individual bypass frustration and are impregnating, as they represent means of self-satisfaction and the capability of autistically finding satisfaction.

Freud’s image of a bird’s egg, with its provision of food inside the shell (Freud, 1911a), can well illustrate these psychotic mechanisms. In schizophrenia, object-cathexes are abandoned through the annulment of preconscious thing-representation: only an intensely invested word-representation remains. However, as the thing-representations inscribed in the unconscious, the primitive object-cathexes have proximity with word-representation, revealing a strange phenomenon in which words and things are equal. In this perspective, in psychosis, the words turn into things, i.e., the psychotic speech is characterized by a concreteness which interferes in the symbolic procedures of communicational language. In summary, this structural weakening of the psychic apparatus splitting, which prevents the distinction and articulation of unconscious thing-representation and preconscious word-representation, worsened by the emptying of preconscious thing-representation, distorts the perception of external reality and, more especially, hampers a socially shared interpretation of this reality. In psychosis, there is commonly an imaginary mold and a concrete understanding of culture’s symbolic net.

It is valid to mention that, in several of Freud’s texts throughout his career, the division of consciousness hypothesis is presented based on an intense affective experience which is incompatible with other representations and cannot be assimilated by the ego, constituting, thus, a situation of trauma. In the article “The Neuro-Psychosis of Defense” (Freud, 1894), the traumatic experience is seen as responsible for the triggering of defense hysteria (*Abwehrhysterie*). Later, after elaborating the idea of death drive, Freud shows in “Beyond the Pleasure Priciple” (Freud, 1920) that the principle of pleasure is in service of death drive, facing trauma as a structural condition of psychism. In “Splitting of the Ego in the Process of Defense” (Freud, 1940), Freud carries on with this theory and argues that psychic conflict produces a splitting of Ego which, in some cases, may lead to a severe loss of reality.

Returning now to the matter of psychosis in *The Unconscious* (Freud, 1915a), one can affirm that the primary process, the free flow of energy, becomes dominant in the way the psychic apparatus functions in psychosis. According to the first pulsional dualism, it can be stated that the psychotic individual is ruled by the principle of pleasure/displeasure, which does not mean that the psychotic experience is drowned in a quest for pleasure, as this would contradict clinical experience. Despite the occasional insinuation of this confusion in Freudian texts, the logic of his theorization is clear: pleasure/displeasure refers to a principle of psychic functioning rather than an affective experience, and, consequently, its predominance or intrusion may (and they frequently do) produce great psychic suffering. This apparent contradiction may evoke a certain perplexity, hence the need to explain the reiteration of suffering. Freud (1920) saw the necessity to define death drive as something that goes “beyond the pleasure principle,” as an extreme and destructive limit to the functioning of the psychic apparatus. Such hypothesis, anticipated in the 1895 “Project for a Scientific Psychology” (Freud, 1895) through the idea of *neuronal inertia* as total energy flow, had been counterbalanced by the idea of constancy, in which the balance of libidinous investments oppose to the tendency to point zero, i.e., the tendency to return to death (?), to the condition of inanimate matter. This initial idea was reconsidered, with a deeper elaboration, when ideas regarding psychosis confronted the theorization of the First Topography.

Therefore, it appears that the *other* (stranger) who frightens the psychotic individual and invades his/her body represents the imaginary image of death drive, and the intense suffering that it produces testifies not the return of what has been repressed, as happens in neurosis, but the threatening and disruptive presence of a pulsating unconscious. Chaotic excitements are mixed with external sources of stimulation, imprisoning the psychotic individual, who has no contact with the stabilizing counterpoint of social reality. This overpowering “pulsating unconscious” presents itself as a profoundly deformed type of alterity, an uncontrollable *other* that does not submit itself to the regulations imposed by the encounter with the other of mediations and symbolic interactions. In fact, the psychotic experience represents a lack of comprehension of the alterity dimension as the recognition of difference built in the process of intersubjective relations.

In the perspective of Freudian psychoanalysis, the psychotic episode happens when the individual is eclipsed; when, facing the frustration of the loss of an object, this individual directs the libido to him/herself, in an effort to desperately invest in his/her ego, which justifies the designation of narcissistic neurosis. In this desperate movement to support themselves, the psychotic and, more especially, the schizophrenic individual, to use the Freudian terminology, are concealed in the auto-erotic circuit of a shattered body. Delusional thinking would have, thus, especially in cases of minor paranoia and its systematic construction, an ordering function, since object-investments would make a re-encounter with the other possible, in a real dimension of alterity which would be represented in and by delusions.

Nonetheless, in the vortex of psychotic episodes emerges the *other*, with no social alterity, the devouring and impersonal presence of a “pulsating unconscious,” a presence of this unrepresented drive which cannot serve as a mold for the disordered excitements and pulsating chaos. Mental automatism, common in psychosis, may be understood as the presence of an *other*, of an exteriority, in the core of a person’s intimacy. The psychotic individual, affected by mental automatism, complains about invasions, abuse, usurpation, voices which scream, and other revealing phenomena of a stranger inhabiting the mind and unsettling the idea of unity and identity.

Therefore, when facing psychotic phenomena through the perspective of Freudian theorization, clinical strategy should consist on introducing symbolic mediation, sometimes represented by the analyst in the institutions, which could offer the psychotic individual an opportunity to reconstruct his/her dimension of alterity. Managing these situations in a context of transferential failure is not at all easy and demands of the analyst the ability to make good use of opportunities of stabilization provided by delusion. In spite of the major difficulties of this task, always taken into account by Freud, it is not unreasonable to conclude that coping with it is a true ethical imperative for the analyst.

## Conflict of Interest Statement

The authors declare that the research was conducted in the absence of any commercial or financial relationships that could be construed as a potential conflict of interest.

## Footnotes

1 The author has used as source for consult a Brazilian edition in which this passage can be found on Freud (1915b, p. 225).
